# Multiple Hamartoma Syndrome with Characteristic Oral and Cutaneous Manifestations

**DOI:** 10.1155/2013/315109

**Published:** 2013-09-16

**Authors:** Prashanthi Chippagiri, Spoorthi Banavar Ravi, Neha Patwa

**Affiliations:** ^1^Department of Oral Medicine and Radiology, MS Ramaiah Dental College and Hospital, Bangalore, Karnataka 560010, India; ^2^Department of Oral Pathology, MS Ramaiah Dental College and Hospital, Bangalore, India; ^3^Department of Dentistry, Indiragandhi District Hospital, Mandsaur, Madhya Pradesh 458001, India

## Abstract

*Aim*. To present a case of Cowden's syndrome and emphasize the importance of continued cancer surveillance in these patients. Cowden syndrome is an inherited autosomal dominant trait with incomplete penetrance and a range of expressivity. It is characterized by multiple hamartomas and neoplasms. Mucocutaneous features include trichilemmomas, oral mucosal papillomatosis, acral keratosis, and palmoplantar keratosis. Here, we report a case of Cowdens syndrome of a 30-year-old female patient who came with a complaint of multiple growths in the oral cavity of a three-month duration. On examination, multiple skin-colored, flat-topped papules over her forehead and right malar bone and multiple papillomatous papules involving all the mucosal surfaces intraorally were observed. This syndrome is associated with the development of several types of malignancies, especially breast carcinoma and thyroid carcinoma, which is why early recognition and regular and vigilant surveillance of individuals with the syndrome are important.

## 1. Background

Cowden syndrome (CS), also termed as multiple hamartoma syndrome, was first described in 1940 by Salem and Steck [1]. Cowden's syndrome was defined and named by Lloyd and Dennis in 1963 referring to their patient Rachael Cowden, who died of breast carcinoma [2, 3]. Weary and coworkers reported 5 more patients in 1972 and suggested the name multiple hamartoma syndrome (MHS) [2, 4].

Cowden's syndrome (CS) is a rare genodermatosis, of autosomal dominant inheritance and variable phenotype, principally characterized clinically by multiple hamartomas of ectodermic, mesodermic, or endodermic origin. It results most commonly from the mutation in the PTEN gene on the arm 10q [5]. Cowden's syndrome is associated with an increased risk of malignancy, particularly cancers of the breast, thyroid, endometrium, and to a lesser extent, renal system [6].

The rare occurrence of this disease and the synchronous association of various malignancies with this disease emphasize the importance of a thorough diagnostic work-up and management of the patients with cowden's syndrome. The present report thus, details the features of a patient who initially presented with oral swellings and was subsequently found to have Cowden's syndrome.

## 2. Case Report

 A 30-year-old female patient came with a complaint of multiple growths in the oral cavity of three months duration. Initially, she had noticed only two growths in the right and left retrocomissural areas. Gradually they increased in number, involving the entire oral cavity. The lesions were not associated with any symptoms. Previously, she was prescribed corticosteroid ointment and multivitamins by a local doctor. She found no relief.

 Her medical history reveals a left breast abscess 12 years back which was drained. She also gave a history of a growth in the left lower back region which was excised 10 years back. However, there were no medical records available for proper evidence. Review of the patient's family history was insignificant.

On general physical examination, she was found to be moderately built and nourished with all her vital signs in the normal range. Pallor was noticed on examination of her inferior palpebral conjunctiva. A scar was noticed over the lower back region on the left side.

**Figure 1:**
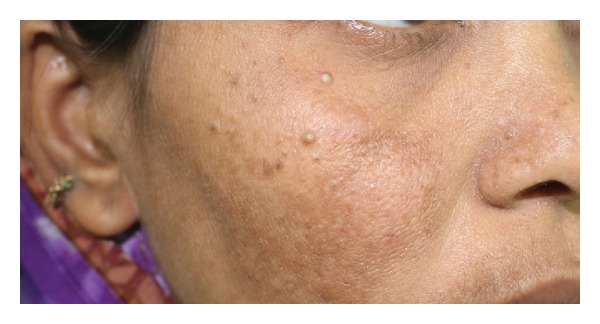
Trichilemmomas on the right malar bone.

 Extraoral examination revealed the presence of small multiple skin colored, flat topped papules over the skin of her forehead and malar bones, measuring around 1 to 5 mm in diameter (Figure 1). The patient was referred to a dermatologist for evaluation of these lesions and they were diagnosed to be trichilemmomas.

**Figure 2:**
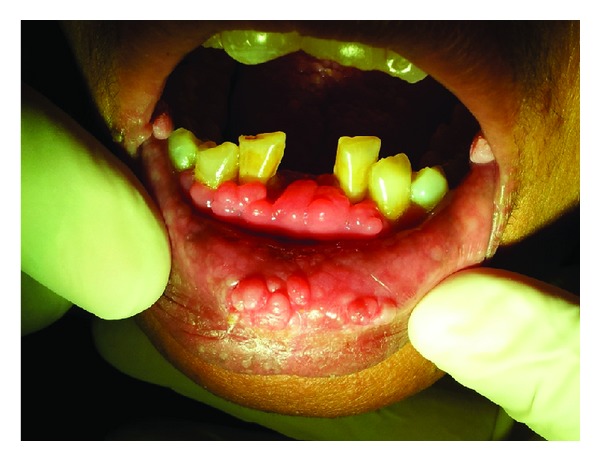
Enlarged gingiva having smooth and multiple papillomatous lesions. Multiple papules may also be noticed on the lower labial mucosa.

**Figure 3:**
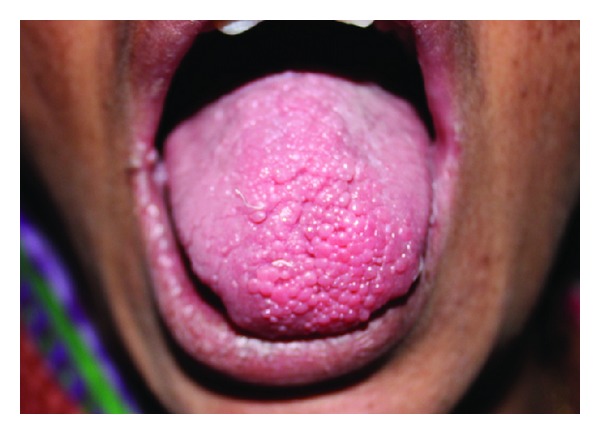
Cobblestone appearance of multiple confluent papules on the tongue.

Intraoral examination revealed multiple papillomatous papules involving all the mucosal surfaces. The presence of multiple, confluent lesions produced a cobblestone appearance. The size of the lesions ranged from 1 to 5 mm. The lesions were spherical and they were coral pink in color (Figures 2 and 3). The lesions on the retrocomissural areas were larger in size and paler. On palpation, the lesions were soft to firm in consistency and nontender. 

## 3. Discussion of the Diagnosis

A provisional diagnosis of Cowden syndrome was made on the basis of the presence of trichilemmomas and mucosal lesions. There were more than 6 cutaneous papules or trichilemmomas and oral papillomatosis. This case, therefore, fulfilled the diagnostic criteria 1 of the International Cowden Syndrome Consortium.

*The International Cowden Syndrome Consortium proposed a set of diagnostic criteria for diagnosis of Cowden's syndrome, which includes the following:*

*pathognomic criteria:*

- facial trichilemmomas
- acral keratosis
- papillomatous papules
- mucosal lesions

*major criteria:*

- breast carcinoma
- thyroid carcinoma
- endometrial carcinoma
- macrocephaly
- Lhermitte-Duclos disease

*minor criteria:*

- thyroid lesions
- mental retardation
- G I Hamartomas
- fibrocystic breast
- lipomas
- fibromas
- genitourinary tumors.

*Operational diagnosis in an individual is based on the presence of any one of the following criteria.*

*Criteria 1*. Mucocutaneous lesions alone if

- 6+ facial papules with 3+ being trichilemmomas,
- cutaneous facial papules and oral mucosal papillomatosis,
- oral mucosal papillomatosis and acral keratosis or,
- 6+ palmar or plantar keratosis.

*Criteria 2.* Two major criteria with one being Lheimitte-Duclos disease or macrocephaly. 

*Criteria 3.* One major and three minor criteria. 

*Criteria 4.* Four minor criteria. 

Orthopantomogram of the patient only revealed generalized alveolar crestal bone loss. Laboratory investigations performed were complete hematological tests and biopsy of the mucosal lesions. Hematological investigation showed the presence of anemia. Biopsy specimen of papules from lower labial mucosa, buccal mucosa, and gingiva showed hyperplastic stratified squamous epithelium with mild inflammatory evidence in the connective tissue suggestive of nonspecific epithelial hyperplasia.

## 4. Treatment and Management

Surgical excision of the lesions present on the right and left retrocommisural areas, the lower labial lesions, and gingivectomy with surgical contouring of the lower anterior gingiva was performed for esthetic reasons.

Owing to the increased risk of these patients to the benign and malignant thyroid diseases and breast and gastrointestinal cancers, thyroid ultrasonography and thyroid function tests and endoscopy for gastric polyps were performed in this patient. The tests revealed no abnormality. The patient was then referred to a gynaecologist for appropriate screening to rule out malignancies of the endometrium and breast. Mammography was performed. No evidence of malignancy was found from the above tests. 

It was decided to follow up the patient closely to monitor the lesions as well as to perform continued cancer surveillance.

## 5. Discussion

Cowden's syndrome is a rare multiple hamartoma disorder [7]. This syndrome is one of a group of heterogeneous disorders known collectively as the *PTEN* hamartoma tumor syndrome (PHTS). Affected individuals usually develop clinical features by their second decade of life [8]; however, there is a considerable heterogeneity in clinical presentation. 

Cowden's syndrome is clinically characterised by multiple mucocutaneous hamartomatous lesions and neoplasias of the breast, thyroid gland, and gastrointestinal tract [2, 9]. Mucocutaneous lesions include flesh-colored flat-topped cutaneous facial papules ranging from 1 to 5 mm in diameter, most of which are trichilemmomas. Multiple oral papules are present on gingival, labial, and palatal surfaces of oral cavity. They measure around 1 to 3 mm, and they coalesce to give a cobblestone appearance to the mucosae. Palmoplantar keratosis is another type of mucocutaneous lesions observed in Cowden's syndrome [10]. Because the development of associated malignancy may take several years, these mucocutaneous lesions may serve as important clinical markers in identifying patients at high risk of malignancy of the breast and thyroid [6].

Women with Cowden's syndrome have a 30% to 50% risk for breast cancer. Therefore, women should carry out monthly self-examinations, and professional physical examinations should be performed once every three months. Mammography is suggested to be performed twice a year. Some authors have recommended prophylactic bilateral mastectomy, particularly in women with an extensive fibrocystic breast disease or breast carcinoma [11, 12].

Thyroid should be examined for any abnormalities. Thyroid function tests and ultrasonography of the gland should be performed as baseline diagnostic examinations. Fine needle aspiration or surgical biopsies are indicated if any lesion is identified. In addition, complete blood cell count, liver and renal function tests can be performed at the baseline [2]. Other investigations which could be carried out are gastrointestinal endoscopy, endometrial examination, radionuclide scans to detect any malignancies, molecular genetic analysis for phosphatase and tensin homolog (*PTEN*), and immunohistochemical staining for PTEN. 

Treatment for Cowden's syndrome is only cosmetic. The facial papules can be treated physically by CO_2_-laser ablation, surgical removal, or chemically removed by topical 5-fluorouracil. Systemic therapy with acitretin 0.75 mg/kg/day has been reported to give good cosmetic results for all mucocutaneous lesions [13]. Surgical management of the mucocutaneous lesions includes cryosurgery, electrosurgery, dermabrasion, and laser abrasion.

## 6. Conclusion

Perhaps, the oral lesions could be the only sign of this disease. Hence, the identification of these lesions will help in early recognition of Cowden's syndrome which may in turn facilitate the early diagnosis of cancer. Hence, frequent follow-ups and continued cancer surveillance is vital for long-term survival of these patients.
